# Manifesto on the Value of Adult Immunization: “We Know, We Intend, We Advocate”

**DOI:** 10.3390/vaccines9111232

**Published:** 2021-10-22

**Authors:** Raffaele Antonelli-Incalzi, Francesco Blasi, Michele Conversano, Giovanni Gabutti, Sandro Giuffrida, Stefania Maggi, Cinzia Marano, Alessandro Rossi, Marta Vicentini

**Affiliations:** 1 Geriatric Unit, Campus Bio-Medico University, 00128 Rome, Italy; R.Antonelli@unicampus.it; 2 Italian Society of Gerontology and Geriatrics, 50129 Florence, Italy; 3 Department of Pathophysiology and Transplantation, University of Milan, 20122 Milan, Italy; francesco.blasi@unimi.it; 4 Respiratory Unit and Adult Cystic Fibrosis Center, Department of Internal Medicine, Fondazione IRCCS Ca’ Granda Ospedale Maggiore Policlinico, 20122 Milan, Italy; 5 Department for Public Health, Local Health Unit Taranto, 74121 Taranto, Italy; michele.conversano@asl.taranto.it; 6 Department of Medical Sciences, University of Ferrara, 44121 Ferrara, Italy; giovanni.gabutti@unife.it; 7 Department of Prevention, Local Health Unit Reggio Calabria, 89124 Reggio Calabria, Italy; sandrogiuffrida@gmail.com; 8 Aging Branch, Institute of Neuroscience, National Research Council, 35128 Padova, Italy; stefania.maggi@in.cnr.it; 9 Medical Affairs, GSK, 1300 Wavre, Belgium; cinzia.x.marano@gsk.com; 10 Italian Society of General Medicine and Primary Care, 50142 Florence, Italy; rossi.alessandro@simg.it; 11 Medical Affairs, GSK, 37135 Verona, Italy

**Keywords:** adults, aging, inflammaging, immunosenescence, Italy, recommendations, vaccination, vaccines

## Abstract

Immunization through vaccination is a milestone achievement that has made a tremendous contribution to public health. Historically, immunization programs aimed firstly to protect children, who were disproportionally affected by infectious diseases. However, vaccine-preventable diseases can have significant impacts on adult mortality, health, and quality of life. Despite this, adult vaccinations have historically been overlooked in favor of other health priorities, because their benefits to society were not well recognized. As the general population is aging, the issue of vaccination in older adults is gaining importance. In high-income countries, recommendations for the routine vaccination of older adults have been gradually introduced. The Italian National Immunization Plan is considered to be among the most advanced adult vaccination plans in Europe. However, available data indicate there is low adherence to vaccination recommendations in Italy. The COVID-19 pandemic has exposed the damage that can be caused by an infectious disease, especially among adults and individuals with comorbidities. The aim of this “Manifesto”, therefore, is to provide an overview of the existing evidence on the value of adult vaccination, in the Italian context, with a call to action to healthcare providers and health authorities.

## 1. Introduction

Immunization through vaccination and safe drinking water have been the two most effective disease prevention measures of all time [1,2]. The first immunization campaigns successfully targeted infectious diseases notorious for their devastating consequences, especially for children [2,3]. The success of these campaigns improved everyday life and gained public support, as the incidence of diseases previously responsible for massive mortality and morbidity dramatically decreased [2,3]. High levels of childhood vaccination coverage were achieved in high-income countries by the late 1950s and in low- to middle-income countries by the 1980s [4]. Historically, therefore, childhood vaccination was the primary focus of societies and health authorities; recommendations for adult vaccinations followed at a much slower pace [5]. Adult vaccination tended to receive less attention because other health priorities were deemed more important and because its benefits to society were not as well recognized [5,6,7,8,9,10]. At the international level, the importance of vaccine-preventable diseases (VPDs) in adults was discussed at a meeting held by the World Health Organization (WHO) in 2017, which concluded that there was a “global mandate” to vaccinate older adults [8]. VPDs have significant impacts on adult mortality, health, and quality of life [9]. In high-income countries, 90% of pneumonia-related deaths and 90% of influenza-induced deaths involve adults aged ≥65 years [10,11]. Chronic diseases in older adults are more difficult to treat in the presence of infectious diseases. Infectious diseases may induce cardiovascular complications, increase the risk of heart disease, and worsen asthma or chronic obstructive pulmonary disease (COPD) symptoms [11,12,13,14,15,16].

Vaccinations prevent about 6 million deaths every year [1], and their efficacy and safety have been widely established [17]. In economic terms, vaccination is one of the most cost-effective health interventions, due to the substantial cost savings and economic growth that it leads to in the long-term [1]. Nevertheless, only a small fraction (<0.5%) of health budgets in many European countries was spent on vaccination in the pre-coronavirus disease 2019 (COVID-19) pandemic era [18].

In high-income countries, recommendations for routine vaccinations in older adults have been gradually introduced [11,19]. Italy has produced a National Immunization Plan 2017–2019, which is considered one of the most advanced adult vaccination plans in Europe [11,20]. However, vaccination coverage data are only available for influenza vaccination, with data on other adult vaccinations scarce; nevertheless, the data that are available all indicate low adherence to vaccination recommendations [21,22,23].

The COVID-19 pandemic has clearly demonstrated the damage that can be caused by an infectious disease, especially among adults and individuals with comorbidities. Although all age groups are at risk of contracting COVID-19, older people face a significantly higher risk of developing severe illness if they become infected with the virus, due to physiological changes associated with aging and potential underlying health conditions [9,24,25]. Chronic diseases that increase the risk of severe COVID-19 had already been identified as underlying risk factors for other infectious diseases [26]. Therefore, the time has come to prioritize adult immunization via vaccination and strengthen the value placed on vaccination among vulnerable populations.

**Figure 1:**
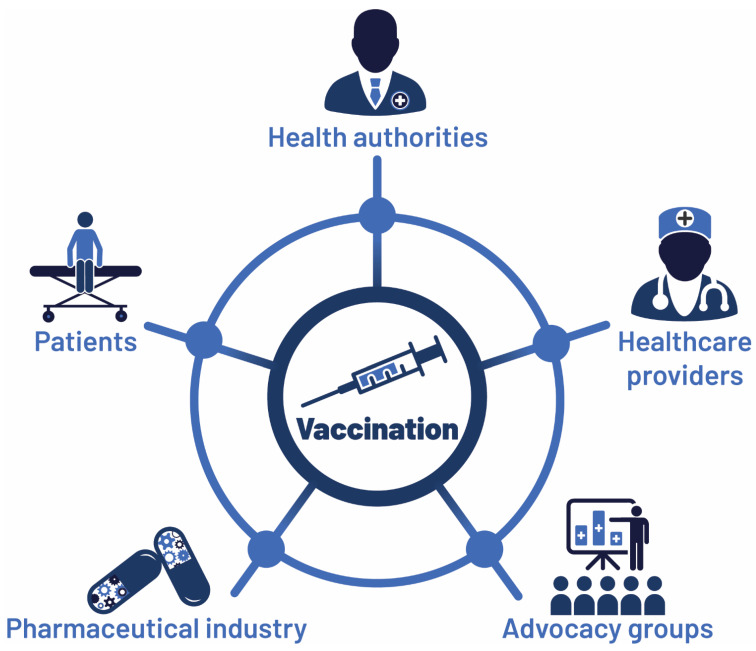
Network of stakeholders involved in the process of vaccination.

The aim of this “Manifesto”, then, is to provide an overview of the existing evidence on the value of adult vaccination, with examples from the Italian context, and present healthcare providers (HCPs) and health authorities with a call to action (Figure 1).

## 2. We Know

### 2.1. The Aging Population, an Epidemiological Shift

According to a 2020 European Commission report on the impact of demographic change in Europe [27], Europeans live their overall long lives in good health. Italians could expect about 60 healthy life-years at birth in 2018 [27]. Compared with other European Union (EU) countries as a whole, with an average life expectancy of 80.6 years, Italy has the second highest life expectancy at birth (82.7 years) after Spain (83.0 years) [28].

Advancements in public health and the subsequent increased life expectancy, combined with a low fertility rate, are skewing the population age structure toward older ages [29,30,31]. In 2019, Italy had the second highest absolute number of adults aged ≥65 years in Europe after Germany and the highest proportion (23%) among the general population, a proportion that is projected to rise to 36% by 2050 [29]. Globally, one in six individuals will be aged ≥65 years in 2050 [29]. In Europe, 30% of the population will be aged ≥65 years by 2070, compared with 20% today [27].

**Figure 2:**
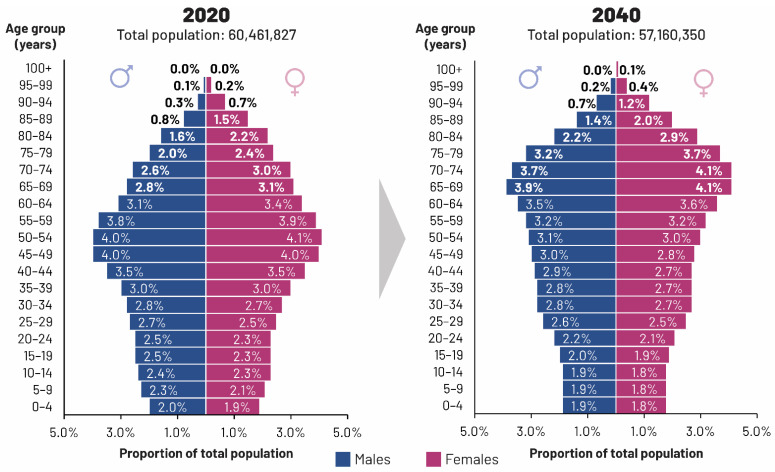
Italian population pyramid, 2020 vs. 2040 estimates. Source: PopulationPyramid.net. Piramide della Popolazione Mondiale dal 1950 al 2100 (World Population Pyramid from 1950 to 2100). Available online: https://www.populationpyramid.net/ (accessed on 19 April 2021) [33] under the terms of the Creative Commons Attribution License (https://creativecommons.org/licenses/by/3.0/igo/, accessed on 10 October 2021). Copyright © 2019 PopulationPyramid.net.

This major demographic and therefore epidemiological shift differs across countries, as the drivers of the change differ in intensity depending on national factors, such as migration, birth, and death rates [27]. Nevertheless, it already has measurable consequences: in Italy, disease burden indicators increased between 1990 and 2017 as a result of population aging [31]. This epidemiological shift is a long-term process: the pattern of the age structure (Figure 2) follows fluctuations in the birth rate, from a high after World War II to a progressively decreasing rate since 1964 [32]. The “baby boom” cohort will continue aging until the 2030s and be gradually replaced within a 30-year period by the cohort of individuals born between 1960 and 1975 that is currently of working age (Figure 2).

**Figure 3:**
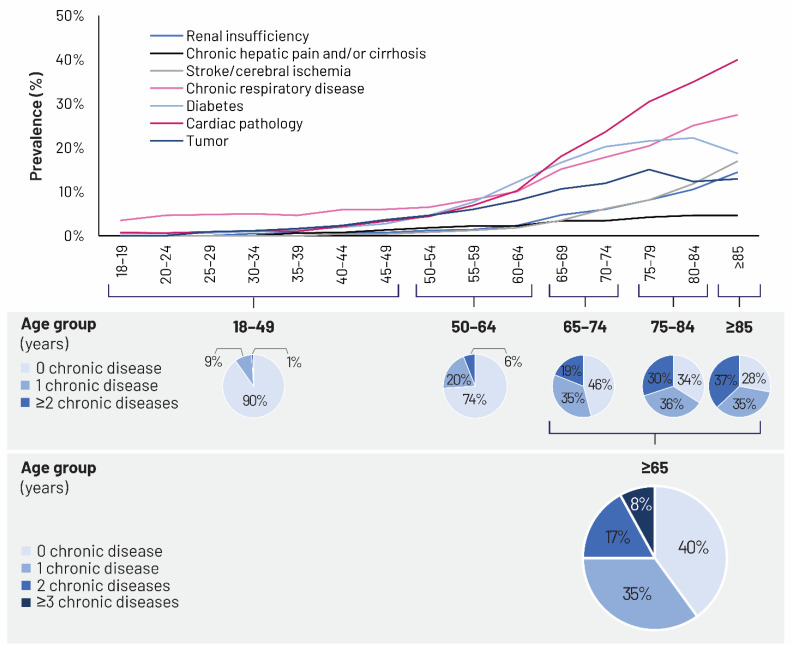
Chronic diseases in the Italian population by age group. Source: Patologie croniche nella popolazione residente in Italia secondo i dati PASSI e PASSI d’Argento (Chronic diseases in the resident population in Italy according to PASSI and PASSI d’Argento data) [35].

As this long-term process of epidemiological shift advances, comorbidities and chronic diseases gain importance with age, and the likelihood of an individual having at least one chronic disease increases [30,34]. The burden of each disease in older age groups should not be considered independently, but in relation to the multifaceted dynamics and interactions of all other comorbidities [30]. These interacting dynamics may result in complex chronic or acute health states, frailty, or acute cognitive decline [30]. The COVID-19 pandemic profoundly exposed the vulnerability of older adults with comorbidities to infectious diseases and highlighted the need for robust healthcare systems to assist them [27]. European data show that 50 million Europeans (mostly ≥65 years of age) suffer from at least two chronic diseases, while chronic diseases account for 70–80% of all healthcare costs [27]. In Italy, a recent analysis estimated that 8.4 million Italians aged ≥65 years live with one chronic disease; this analysis, coordinated by the National Institute of Health, was based on 2015–2018 data on chronic diseases recorded by the surveillance systems “Progresses in Health in the Italian Local Health Units” (PASSI, Progressi delle Aziende Sanitarie per la Salute in Italia; dedicated to the population aged 18–69 years) and PASSI d’Argento (“Silver Steps”; dedicated to the population aged ≥65 years) [35]. According to the same analysis, more than 50% of Italians aged 65–74 years and about 75% of Italians aged ≥85 years live with at least one chronic disease. At the same time, 37% of Italians aged ≥85 years live with at least two chronic diseases (Figure 3) [35]. The most frequent chronic disease is heart disease, followed by chronic respiratory disease, diabetes, cerebral ischemia, renal insufficiency, tumors, and liver cirrhosis (Figure 3) [35]. In Italy, cardiovascular diseases are also the leading cause of death, followed by cancer. Based on 2014 data, 40% of deaths in women and 33% in men had cardiovascular etiology; the corresponding figures for cancer were 24% and 33% [28]. Population aging has also increased the burden of Alzheimer’s disease and other types of dementia, as indicated by the 77.9% increase in disability-adjusted life-years (DALYs) between 1990 and 2017 [31]. During the same period, large increases in DALYs were also recorded for pancreatic (39.7%) and uterine cancers (164.7%) [31]. There has been an increasing trend in the prevalence of chronic diseases over recent years, particularly among older adults aged ≥65 years. In the 65–84-years age group, from 2000 to 2013, diabetes increased from 12% to 17%, hypertension from 35% to 48%, and heart attacks from 4% to 6% [36].

### 2.2. Aging, Immunosenescence, and Chronic or Infectious Diseases

**Figure 4:**
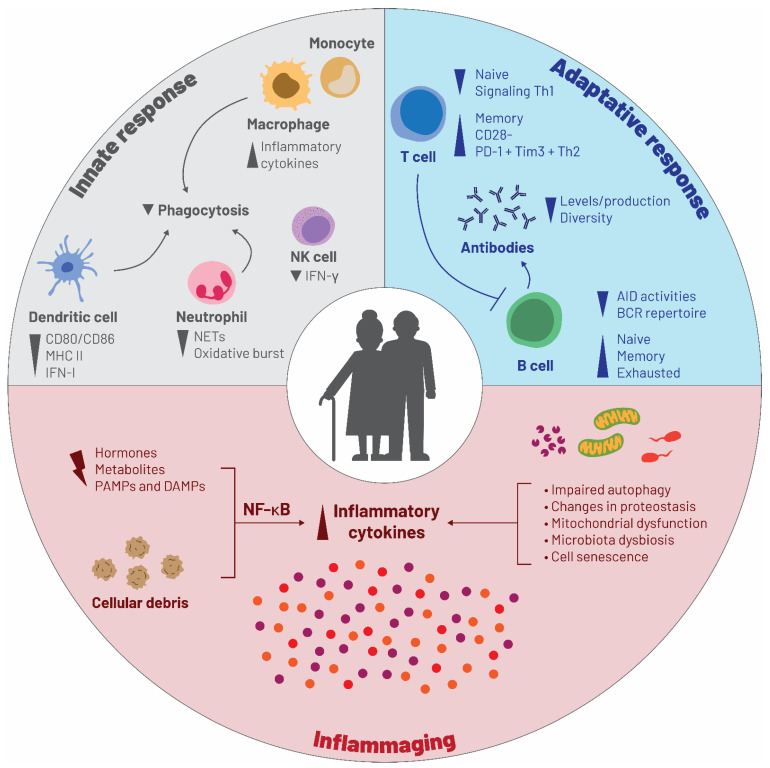
Aging, immunosenescence, and chronic or infectious diseases. Abbreviations: AID, activation-induced cytidine deaminase; BCR, B cell receptor; CD, cluster of differentiation; DAMP, damage-associated molecular pattern; IFN-I, interferon type I; IFN-γ, interferon gamma; MHC, major histocompatibility complex; NET, neutrophil extracellular trap; NF-κB, nuclear factor kappa B; PAMP, pathogen-associated molecular pattern; PD-1, programmed cell death 1; Th, T helper cell; Tim3, T-cell immunoglobulin and mucin-domain containing-3. Source: Adapted from Figure 1 of the article “I mmunosenescence and Inflammaging: Risk Factors of Severe COVID-19 in Older People” (https://doi.org/10.3389/fimmu.2020.579220, accessed on 10 October 2021) by Anna Julia Pietrobon, Franciane Mouradian Emidio Teixeira, and Maria Notomi Sato, *Front. Immunol.* **2020**, *11*, 579220 [41], available under the terms of the Creative Commons Attribution License (http://creativecommons.org/licenses/by/4.0/, accessed on 10 October 2021). Copyright © 2020 Pietrobon, Teixeira and Sato.

The human immune system continually changes with age [37]. Multiple gradual biological alterations (Figure 4) affect the innate and adaptive immune systems and progressively reduce their functional ability to mount an effective antibody response, a condition known as “immunosenescence” [30,38,39]. Chronological aging per se, i.e., aging of the brain, heart, lung, as well as musculoskeletal, gastrointestinal, and other body systems, can also impact the cells involved in the innate and adaptive immune response (Figure 4) [40]. Apart from age, other external environmental factors, such as personal history of responses to pathogens, underlying medical conditions, and most importantly chronic, low-grade inflammation associated with age (referred to as “inflammaging”), are all important contributors to immunosenescence [40,41]. Inflammaging is induced by stress and continuous antigen stimulation [42]. These processes gradually increase the levels of serum inflammatory mediators, such as interleukin-6 (IL-6), IL-1, IL-1RA, tumor necrosis factor-α, and C-reactive protein [41]. Eventually, the chronic persistence of low levels of inflammatory cytokines favors susceptibility to diseases because it facilitates the reaching of a pro-inflammatory threshold, above which a disease or other disability manifests [42]. Moreover, intracellular modifications of senescent cells during inflammaging can activate signaling pathways that further increase the secretion of cytokines, chemokines, and growth factors [41]. The increased levels of pro-inflammatory cytokines are in turn associated with diseases of older adults, such as the neurodegenerative diseases dementia and Parkinson’s disease, chronic heart failure, atherosclerosis, and type 2 diabetes [43,44,45,46,47].

Inflammaging is a major contributor to many chronic diseases associated with aging and acts as a trigger for the clinical manifestation of the respective underlying pathologies [47]. Therefore, immunosenescence has been associated with an increased likelihood of cancer, diabetes, and COPD, as well as infectious, autoimmune, neurodegenerative, and cardiovascular diseases [40,48,49,50,51]. The age-related decline in immune system responsiveness through immunosenescence leads to increased susceptibility to infections in older adults [52]. Furthermore, the severity and medical sequelae of infectious diseases, such as influenza, herpes zoster (HZ), pertussis, pneumococcal disease, and COVID-19, increase with age [9,52,53,54].

### 2.3. The Unfavorable Link between Infectious and Chronic Diseases

We have now established that a number of infections are determinants of chronic diseases [55]. Moreover, chronic conditions can originate from infections [56]. For example, cervical cancer, the fourth most common cancer among women, is caused by the human papilloma virus (HPV) [57]; hepatitis B (HepB) and C viruses can cause hepatocellular carcinoma [58]; and human immunodeficiency virus infection has become a new chronic disease [59,60].

Infectious diseases, regardless of age, can increase the severity of underlying pre-existing chronic diseases and lead to functional decline, loss of autonomy, disability, or death [55]. For example, seasonal influenza infection in people with pre-existing circulatory or respiratory disease may result in their hospitalization or death [61]. Adults aged more than 50 years and who have HZ are at increased risk of asthma, heart disease, stroke, and transient ischemic attack [12,13,62,63,64], while pertussis infection is likely to increase the severity of symptoms in patients with pre-existing asthma or COPD [14,15,16].

### 2.4. Vaccine-Preventable Diseases and Vaccination Benefits

The efficacy and safety of vaccines is well established and it has been clearly proven that, with an excellent safety profile, vaccines can help to control, eliminate, and even eradicate VPDs [1]. Vaccinations have achieved reductions in the incidence of most childhood VPDs to such low levels that the severe manifestations and complications associated with these diseases are no longer remembered [17]. The eradication of smallpox, a devastating infectious disease caused by the variola virus, and which had afflicted humans for more than 3000 years, was a landmark achievement [65]. It is hoped that poliomyelitis, caused by poliovirus, will also soon be under control [1,66]. High vaccination coverage (>95%) has the potential to eliminate measles in most parts of the world, while combined measles, mumps, and rubella (MMR) vaccines could probably also eradicate or at least control rubella and mumps [1]. Furthermore, in vaccinated individuals, infectious diseases have milder manifestations, shorter duration, and fewer complications [1]. Most vaccines primarily prevent disease. Others, such as hepatitis A (HepA) and HPV vaccines, offer protection against both disease and infection, at a level of 90% for HepA and 100% for HPV; this is known as “sterilizing immunity” [1]. The benefits of vaccination extend well beyond the prevention of VPDs in individuals and can protect society as a whole [1]. Once a certain number of individuals in a population has been vaccinated against a particular pathogen (this number can vary according to the specific pathogen and other factors), the spread of the pathogen can be halted, a phenomenon known as ‘herd immunity’ [1].

In older adults in particular, vaccinations can protect them from infectious diseases that have difficult to manage medical complications, require long-term treatments with an associated risk of adverse events, and result in an overall functional decline [67]. It is also important for this age group that vaccination can reduce polymedication and improve medication adherence [67]. Furthermore, vaccinations can increase longevity, protect against secondary diseases and certain cancers, as well as decrease the morbidity and burden induced by chronic illness [1,67,68]. Evidence suggests that vaccination can reduce hospital stays, work absences, and dependence on others [67,69]. Influenza vaccination of individuals aged ≥65 years in the United States (US), for example, was associated with a 27% reduction in the risk of hospitalization for pneumonia or influenza (odds ratio [OR] 0.73; 95% confidence interval [CI], 0.68–0.77) and a 48% reduction in the risk of death (OR 0.52; 95% CI, 0.50–0.55) [70]. Dual influenza and pneumococcal vaccination of >48,000 individuals aged >60 years in China was associated with a decreased risk of hospitalization for acute respiratory illness (OR 0.49; 95% CI, 0.41–0.59) [71]. A recent meta-analysis of observational studies published up to 2019 found that, in patients with diabetes, influenza vaccination was associated with a lower mortality rate (OR 0.54, 95% CI, 0.40–0.74) [72]. The risk of hospitalization due to pneumonia was also lower among the vaccinated individuals (OR 0.89; 95% CI, 0.80–0.98) [72]. Among patients with COPD, influenza vaccination significantly reduced exacerbations (weighted mean difference 0.37; 95% CI, 0.64–0.11; *p* = 0.006) [73]. Pneumococcal vaccination also reduces COPD exacerbations and protects patients against community-acquired pneumonia [74]. Moreover, data suggest that pertussis vaccination can help to prevent symptoms worsening in adults with pre-existing asthma or COPD [16] and block transmission from adults to vulnerable populations such as unvaccinated neonates [54]. It is also suggested that the prevention of HZ through vaccination may reduce the risk of stroke, transient ischemic attack, and myocardial infarction [12,13,63].

Increased adult vaccination coverage rates would have the additional benefit of helping in the fight against antimicrobial resistance (AMR) [75,76,77]. AMR is a major problem, especially for Italy, which is among the European countries with the highest rate of drug-resistant isolates; for example, there is a high prevalence of gram-negative bacteria resistant to multiple antibiotics in intensive care units across Italy [78]. There is also a high incidence of intubation-associated pneumonia and catheter-associated bloodstream infections [78], posing serious treatment challenges for patients resistant to last-line antibiotics [79]. WHO has issued a global action plan to tackle AMR, and this plan includes the use of vaccinations [76]. Vaccinations can help to prevent antibiotic resistance [1] in various ways. They induce antibody-mediated destruction of pathogens and reduce the number of individuals who require antibiotics, whether in vaccine recipients or other individuals protected through herd immunity [80,81,82]. This also leads to reduced opportunities for pathogens to proliferate and, therefore, resistant strains are less likely to develop [75,82]. In a single-blinded placebo-controlled study in Italy, 13.2% (*p* < 0.001) fewer courses of antibiotics were administered to children with a history of acute otitis media who had previously been vaccinated against influenza [83]. In the US, pneumococcal vaccination of adults aged >65 years was associated with a 49% reduction in penicillin-resistant *Streptococcus pneumoniae* strains [84]. In a retrospective analysis of primary care data in the United Kingdom, older adults (aged ≥65 years) who had received an influenza vaccine were 14% less likely to be given antibiotics than their non-vaccinated counterparts [85].

### 2.5. Vaccination Recommendations

**Figure 5:**
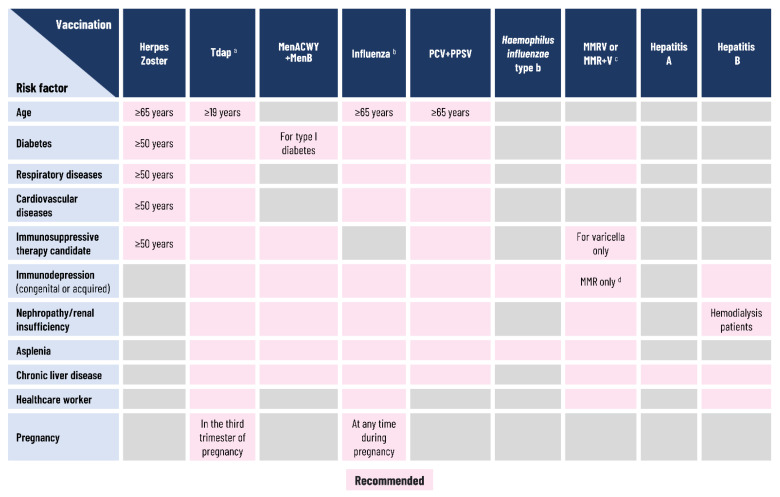
Recommended vaccinations for Italian adults. ^a^ One dose every 10 years; ^b^ One dose per year; ^c^ Susceptibility of adults to these diseases should be checked and vaccination offered if susceptible. Susceptible women should be vaccinated ≥1 month before pregnancy; ^d^ Immunosuppression with CD4 lymphocyte count ≥200/mL. Abbreviations: CD4, cluster of differentiation 4; MenACWY, meningococcal vaccine against meningococcal disease due to serogroups A, C, W, and Y; MenB, meningococcal vaccine against meningococcal disease due to serogroup B; MMR, measles, mumps, and rubella vaccine; MMRV, measles, mumps, rubella, and varicella vaccine; PCV, pneumococcal conjugate vaccine; PPSV, pneumococcal polysaccharide vaccine; Tdap, tetanus toxoid, reduced diphtheria toxoid, and acellular pertussis vaccine; V, varicella vaccine. Source: Piano Nazionale Prevenzione Vaccinale 2017–2019 (National Immunization Plan 2017–2019) [20].

Most high-income countries, including Italy (Figure 5), have developed recommendations for adult (≥19 years) vaccinations [8,19,20,86,87,88,89]. The Italian Ministry of Health recommends the periodic vaccination (every 10 years) of all adults, as well as all women during each pregnancy, with the combined tetanus toxoid, reduced diphtheria toxoid, and acellular pertussis (Tdap) vaccine, as well as MMR and varicella vaccination for adults who have no history of contracting the diseases or relevant previous vaccinations [20]. Annual influenza vaccination is recommended for any adults aged 19–64 years if they are in a risk group and for all adults aged ≥65 years; the ultimate goal is to expand this latter recommendation over time to all those aged ≥50 years [20]. In 2020–2021 and 2021–2022, influenza vaccination recommendations have been extended to include adults aged 60–64 years, to reduce complications and hospitalizations, as the co-circulation of influenza and severe acute respiratory syndrome coronavirus 2 (SARS-CoV-2) viruses could not be excluded [90,91]. For all adults ≥65 years, pneumococcal and HZ vaccinations are also recommended (Figure 5) [20]. HepA and pneumococcal vaccinations are recommended for at-risk adults, and HZ vaccination is recommended for at-risk individuals aged ≥50 years [20]. The quadrivalent meningococcal vaccine against serogroups A, C, W, and Y is also recommended for adults in cases of persisting epidemiological risk [20].

For women of childbearing potential, the recommendations include having the MMR, varicella, Tdap, and HPV vaccines, while it is recommended that pregnant women are vaccinated against influenza and pertussis [92]. Several scientific societies have endorsed the recommendations for women of childbearing potential and pregnant women, including the Italian Society of Obstetrics and Gynecology (SIGO, La Società Italiana di Ostetricia e Ginecologia) [93], the Italian Society of Pediatrics (SIP, Società Italiana di Pediatria), the Italian Society of Hygiene, Preventive Medicine and Public Health (SItI, Società Italiana di Igiene, Medicina Preventiva e Sanità Pubblica), the Italian Federation of Pediatricians (FIMP, Federazione Italiana Medici Pediatri), and the Italian Federation of General Practitioners (FIMMG, Federazione Italiana Medici di Medicina Generale) [94]. For patients with diabetes, evidence suggests they have an increased likelihood of contracting and dying from an infectious disease. Therefore, the scientific associations FIMMG, SItI, the Italian Association of Medical Diabetologists (AMD, Associazione Medici Diabetologi), the Italian Society for the Study of Diabetes (SID, Società Italiana di Diabetologia), and the Italian Society of General Medicine and Primary Care (SIMG, Società Italiana di Medicina Generale e delle Cure Primarie) recommend influenza, pneumococcal, Tdap, HZ, and meningococcal vaccinations for all patients with diabetes [95]. Lastly, for patients with COPD, the Global Initiative for Chronic Obstructive Lung Disease (GOLD) recommends influenza and pneumococcal vaccinations to decrease these patients’ risk of lower respiratory tract infections and Tdap vaccination to protect previously unvaccinated adults against pertussis, tetanus, and diphtheria [74].

As a result of the COVID-19 pandemic, several scientific associations have updated their vaccination recommendations [74,96,97]. In Italy, SIP, SITI, FIMP, and FIMMG stress that it is more urgent than ever to increase protection for all diseases that are likely to affect people at risk of severe COVID-19, and further worsening the health status of the most fragile population [97]. Therefore, influenza, pneumococcal, Tdap, and HZ vaccinations should be provided for older adults [97].

### 2.6. Surveillance of Infectious Diseases in Italy

Despite notification of VPDs being compulsory in Italy, cases are reported on a voluntary basis, and underreporting is common [98,99,100]. The epidemiological value of surveillance systems is impaired due to their low sensitivity in distinguishing real epidemiological changes in diseases from system artifacts [98]. In Italy, a national network for a sentinel surveillance system has only been established for pediatric cases of measles, mumps, rubella, pertussis, and varicella [101]. For all other cases of confirmed or suspected infectious diseases, a passive surveillance system is used [100]. This system is based on reports from physicians, who must report each case and related information to their local health unit, which in turn reports to the regional health authority, from where data are sent to the Ministry of Health and the National Institute of Statistics [100,101,102]. Following an increase in bacterial infections in 2015, updated specific standard procedures were issued in 2017 requiring more attention to be paid to the diagnosis of bacterial diseases and improved timelines for the reporting of invasive bacterial diseases caused by *Neisseria meningitidis*, *Streptococcus pneumoniae*, and *Haemophilus influenzae* [103,104].

### 2.7. Vaccination Coverage

The Italian National Immunization Plan has set vaccination coverage objectives, based on which the coverage rates by 2020 for pneumococcal and HZ vaccination among those aged ≥65 years should reach 75% and 50%, respectively [105]. The vaccination coverage target for influenza vaccination was set at a minimum of 75%, particularly for citizens aged ≥65 years, and an optimum of 95% [106].

Unfortunately, with the exception of influenza vaccination, there are no data available for adult vaccination coverage in Italy [98,106,107,108]. The data available for influenza vaccination show that just 16.8% of the general population and 54.6% of those aged ≥65 years received seasonal influenza vaccination in 2019–2020 [106].

The recent COVID-19 pandemic highlighted the need to increase vaccination coverage rates [97]. However, there is also a need to develop reliable vaccination registries. These would allow identification of unvaccinated or under-vaccinated individuals, monitoring of the implementation of immunization programs, and help to improve coverage of underserved populations [109].

### 2.8. Vaccination Barriers

In Italy, after decades of trust and public confidence in the value of vaccines, a growing phenomenon of vaccine hesitancy has severely impacted vaccination coverage rates, even among traditionally well-accepted vaccines (poliomyelitis, diphtheria, tetanus, HepB, pertussis, *H*. *influenzae* type b) [94,110]. To combat this trend, the Italian Ministry of Health enacted a law in 2017 that require that children entering the Italian educational system have received poliomyelitis, diphtheria, tetanus, HepB, pertussis, *H. influenzae* type b, MMR, and varicella vaccinations [111].

The reduced public confidence in vaccines and the rise of vaccine hesitancy is, unfortunately, a global phenomenon that is threatening to undo the progress made against infectious diseases; for this reason, vaccine hesitancy was included in the WHO list of global threats to health in 2019 [112]. Healthism, misinformation, and hoaxes, as well as skepticism toward science, all contribute to reduced confidence in vaccines [113].

In the case of barriers to adult vaccination, there is the additional factor of limited public awareness of the existence of adult vaccination recommendations, which unfortunately is coupled with limited awareness of the subject among HCPs [7]. Access to free, accurate information, and increased HCP awareness and preparedness regarding vaccinations, would help to restore vaccine confidence [113]. Information gaps are evident from the low levels of knowledge among HCPs about older adult vaccinations [114]. Furthermore, HCPs give a lower priority to the vaccination of adults compared with their emphasis on other preventive measures and, as a result, often fail to recommend vaccination [7]. Therefore, there is a clear need to address these knowledge gaps among both the public and HCPs, in terms of adult vaccination recommendations for the general population and for individuals with specific chronic conditions [7]. A life-long approach to vaccination is needed among HCPs and the public, as well as the inclusion of adult immunization in vaccination priorities [11,115]. This will soon become imperative upon wider realization of the ongoing demographic shift toward older ages [115]. Furthermore, the impact of any vaccination campaigns should be monitored, and there is also a need for vaccination registries and disease surveillance. HCPs should be engaged in these processes, while efforts should be made to increase their awareness of vaccination strategy goals and the importance of disease surveillance [99]. Automatic reminders for HCPs could facilitate and improve the surveillance process [99]. Registering a case or a vaccination should be simplified and unified across the country [99].

We need to stimulate research and increase resources to address the unmet needs and interventions necessary for the benefit and well-being of people in the second half of their life [116]. Research is also required to identify the main barriers that result in suboptimal vaccination coverage with the recommended vaccinations in each age group and determine the best approaches to accelerate vaccination in each of these age groups. The importance of basic and clinical research and their interconnection with new technologies, resources, vaccines, and, ultimately, political decisions involving the general population was clearly demonstrated with the emergence of the COVID-19 pandemic. Just five days [117] following the sequencing of the SARS-CoV-2 virus in January 2020 [118], research toward the development of vaccines against COVID-19 began, which resulted in the first vaccines being approved in December of the same year [119]. Administered as part of mass vaccination programs, real-world effectiveness data have shown that the newly produced vaccine is 90% effective in preventing new infections [120], which caused a dramatic decrease in incidence and hospitalizations [121]. As a result of this experience, large swathes of the public have come to understand that the results of scientific research are essential for the successful management of health emergency situations and also to inform decision-makers’ actions [122,123].

## 3. We Intend

The need to prioritize adult vaccination, as exemplified by the existing evidence, must be transformed into action plans with clear responsibilities, targets, and goals for stakeholders (Figure 1). Each type of stakeholder can act according to a specific action plan toward achieving an adequate number of adults participating in immunization through vaccination. Table 1 outlines calls to action for each group of stakeholders.

## 4. We Advocate

All stakeholders involved in the process of vaccination should work together to ensure that people live long and healthy lives (Figure 1). The benefits of vaccination should become a major and ongoing topic of conversation beyond the current pandemic context. We advocate for the implementation of a paradigm shift, in which citizens eligible for immunization are proactively identified and informed from HCPs about relevant vaccines before they become sick.

## Figures and Tables

**Table 1:** Calls to action for each group of stakeholders involved in the process of vaccination.

**Health Authorities and Health Institutions**
⚬ Increase investment in research and new technologies. ⚬ Improve epidemiological reporting systems for infectious diseases. ⚬ Implement and harmonize immunization registers to collect reliable coverage data. ⚬ Provide HCPs and the public with access to immunization registers. ⚬ Provide HCPs and the public with simple tools to inform them of their vaccination needs, such as a vulnerability index and algorithms that help HCPs to identify vaccination needs among their patients. ⚬ Establish systems for tracking adults, to enable follow-up and issue reminders about necessary vaccinations. ⚬ Improve access to vaccination. ⚬ Establish education and training programs for physicians and other HCPs; patients’ associations should also be involved.
**HCPs**
⚬ Increase HCPs’ awareness and education around infectious diseases. ⚬ Add immunization as a regular topic in the educational programs of relevant scientific societies. ⚬ Ensure a minimum level of training for HCPs on the benefits and availability of vaccines to the general population. ⚬ Encourage networking among specialists and general practitioners to improve patient follow-up (a multidisciplinary approach to guide patients). ⚬ Ensure that discussions about vaccination are part of a routine visit to the doctor and are included in Diagnostic Therapeutic Assistance Pathways.
**Public**
⚬ Increase awareness of the benefits of vaccination, both among the general population and high-risk patients. ⚬ Provide, in lay terms, accurate information from trustworthy, comprehensive, and accessible sources. ⚬ Involve patients’ associations in discussing patient needs and in creating appropriate content to communicate information about vaccination. ⚬ Improve the health and scientific literacy of the population, e.g., through educational programs for the lay public. ⚬ Include both scientific and lay testimonials to effectively engage the public with a unique message. ⚬ Develop specific disease awareness campaigns aimed at the general public.
**Pharmaceutical Industry**
⚬ Better and more timely communication of scientific data on the efficacy and safety of vaccines. ⚬ Improve the communication around vaccine development and manufacturing to increase public trust in vaccines. ⚬ Support independent research, educational initiatives, and disease awareness campaigns.

Abbreviations: HCP, healthcare provider.

## Data Availability

Data sharing not applicable to this article as no datasets were generated or analyzed during the current work.

## Abbreviations

AMD	Italian Association of Medical Diabetologists
AMR	antimicrobial resistance
CI	confidence interval
COVID-19	coronavirus disease 2019
COPD	chronic obstructive pulmonary disease
DALY	disability-adjusted life-year
EU	European Union
FIMMG	Italian Federation of General Practitioners
FIMP	Italian Federation of Paediatricians
HCP	healthcare provider
HepA	hepatitis A
HepB	hepatitis B
HZ	herpes zoster
HPV	human papilloma virus
MMR	Measles, mumps, and rubella
OR	odds ratio
PASSI	Progresses in Health in the Italian Local Health Units
SID	Italian Society for the Study of Diabetes
SIGO	Italian Society of Obstetrics and Gynecology
SIMG	Italian Society of General Medicine and Primary Care
SIP	Italian Society of Pediatrics
SItI	Italian Society of Hygiene Preventive Medicine and Public Health
Tdap	tetanus toxoid, reduced diphtheria toxoid, and acellular pertussis
US	United States
VPD	vaccine-preventable disease
WHO	World Health Organization
